# Organic Eu3+-complex-anchored porous diatomite channels enable UV protection and down conversion in hybrid material

**DOI:** 10.1080/14686996.2020.1799693

**Published:** 2020-10-28

**Authors:** Xiaoshuang Yu, Lili Li, Yue Zhao, Xinzhi Wang, Yao Wang, Wenfei Shen, Xiaolin Zhang, Yanying Zhang, Jianguo Tang, Olle Inganäs

**Affiliations:** a Institute of Hybrid Materials, National Center of International Joint Research for Hybrid Materials Technology, National Base of International Sci. & Tech. Cooperation on Hybrid Materials, Qingdao University, Qingdao, P. R. China; b Biomolecular and Organic Electronics, Department of Physics, Chemistry and Biology (IFM), Linköping University, Linköping, Sweden

**Keywords:** Organic Eu^3+^-complex, diatomite, surface modification, luminescent hybrid material, PET film, transmittance, 206 Energy conversion, transport, storage, recovery

## Abstract

The organic Eu^3+^-complex [Eu(TTA)_3_Phen] has been incorporated into the channels of surface-modified frustules from diatoms as a key material to absorb and convert UV-photons to visible luminescence. Systematic investigation results indicate that the organic Eu^3+^-complex encapsulated in the functionalized diatomite channels exhibits enhanced luminescence and longer lifetime, owning to the Eu(TTA)_3_Phen complex interacting with its surrounding silylating agents. The organic Eu^3+^-complex-anchored porous diatomite hybrid luminescent material was compounded with polyethylene terephthalate (PET) by using a mini-twin screw extruder to prepare a self-supporting film of the hybrid material. Besides, the UV absorption properties of the composite films were investigated. These films will potentially be related to the UV protection of photovoltaic devices.

## Graphical abstract

**Figure:**
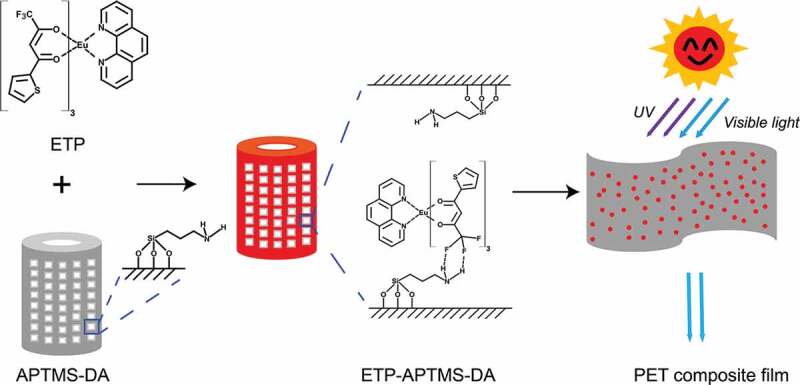

## 1. Introduction

Ultraviolet (UV) light reaches the surface of the Earth after passing through the stratospheric ozone layer [1–3] causing the degradation and aging of organic materials, especially polymer materials including films and fibres. Furthermore, degradation and aging result in deterioration of mechanical properties and the optical properties. Therefore, it is demonstrated that avoiding UV light plays an important role in protecting the polymer materials. It has been found that the photovoltaic devices used outdoors usually encounter UV damaging problem. With the development of polymer solar cells, the efficiency of single-junction polymer solar cells has now been over 16%, which is much close to the commercial application level [4]. However, the weak stability of polymer solar cells becomes the critical obstacles for commercial applications outdoors, because the active layer materials of the photovoltaic devices suffer from damage under UV radiation reducing photovoltaic performance [5,6]. Therefore, it is essential to protect active polymer materials in polymer solar cells from UV light radiation, especially UV-A (the wavelength range is 400–315 nm) and UV-B (the wavelength range is 315–280 nm) in solar radiation.

Thus, researchers have developed different UV-protection agents. For example, the cotton fabric modified with ZnO nanoparticles on the surface offers UV-blocking in the wavelength range from 280 nm to 400 nm [7,8]. Prosa et al. have improved the stability of polymer solar cells under UV radiation by doping ZnO layer with aluminium [6]. On the other hand, as a conventional polymer material, polyethylene terephthalate (PET) is one of the best choices as flexible substrates due to its features such as low cost, easy synthesis, high transparency and excellent stability [9]. Some studies focused on the films against UV used outdoors based on PET with the inclusion of ZnO [10–12]. Gheno et al. prepared organic and perovskite solar cells with improved performance by utilizing the materials absorbing UV light [13,14].

Recently, designing visible luminescence materials by lanthanide complexes has attracted increasing attention for various applications [15,16]. Europium(III) ion has abundant energy levels and unique 4 f electronic transitions that result in high-intensity photoluminescence (PL) and efficient quantum yield (Φ_tot_) [17]. In order to achieve high-intensity PL, 1,10-phenanthroline (Phen) and 2-thenoyltrifluoroacetone (TTA) were introduced as the ligands to produce a complex, Eu(TTA)_3_Phen (ETP), with wide optical absorption [16–20]. Under UV radiation, this complex absorbs the energy and emits bright red light through relaxing the excited electrons in ligand (TTA) and transferring the energy to Eu(III) ions, which is known as the ‘antenna effect’ [17,19,20]. In addition, the ETP complex has been reported to be an excellent luminescence material absorbing UV-A (315–400 nm) and UV-B (280–315 nm) [16,17,21]. Based on the facts mentioned above, ETP was chosen as UV absorber in this work. It has been reported that the ETP complex can be doped to mesoporous silica materials, such as MCM-41, FDU-1 and SBA-15 [19,20,22–24]. However, these mesoporous silica materials are expensive and their production process is not environmentally friendly [26,27]. Therefore, developing the novel mesoporous silica materials is important. Diatomite (DA), is a natural amorphous silica biomineral with hierarchically ordered mesoporous structure [28]. DA is found in sediments of diatoms. Researchers have indicated that the structure of diatom can filter out UV radiation and thus can protect DNA [21,28]. Based on its unique physical and chemical advantages including high specific surface area, high porosity, and chemical inertness, DA has been widely applied in bone growth, filter media and adsorbent [28–31]. Additionally, the surface of diatomite contains abundant silicon hydroxyl groups (Si-OH), which can be physically bonded by ETP complex [19,20,23,32,33]. According to reports, utilizing silane coupling agents is a useful way to improve the physical and chemical properties of the DA surface via modifications [27,34].

In this work, the ETP complex was anchored in the pores of DA via the grafted (3-aminopropyl) trimethoxysilane (APTMS) through impregnation method, which improves the photophysical properties of the complex. Compared with the pure ETP complex, the luminescence properties of the sample encapsulated in APTMS-modified DA are greatly improved. Moreover, the existence of APTMS is much helpful to improve the luminescence properties of the organic Eu^3+^-complex. More importantly, this organic-inorganic hybrid luminescent material was applied as UV absorbance additive for PET composite film, and exhibited excellent UV-protection properties.

## 2. Experimental section

### 2.1. Materials and chemicals

Diatomite (DA) was obtained from Lumino food grade diatomaceous earth (Vancouver, WA, USA). (3-Aminopropyl) trimethoxysilane (APTMS, 97%) was purchased from Shanghai Macklin Biochemical Technology Co., Ltd. Ammonium hydroxide (NH_3_•H_2_O, 25%), toluene (99.5%) and ethanol (95%) were purchased from Sinopharm Chemical Reagent Co., Ltd. Europium chloride hexahydrate (EuCl_3_•6H_2_O, 99.99%), 1,10-phenanthroline (Phen, 99%) and 2-thenoyltrifluoroacetone (TTA, 98%) were purchased from Shanghai Aladdin Biochemical Technology Co., Ltd. Polyethylene terephthalate (PET) was purchased from Sinopec Yizheng Chemical Fiber Co., Ltd.

### 2.2. Purification of raw DA material

The purified process of the raw DA materials [35] is as follows: 5.0 g of the raw DA material was added in 100 ml deionized water, sonicated for 15 min, and collected the particles that settled first and removed the lighter suspended particles (including shattered diatomite parts and the impurities) with the help of a burette to separate the intact diatomite. After five cycles of the washing process (without sonication), the DA samples were dried in a vacuum drying oven.

### 2.3. Modification of DA

2 mL of APTMS was added in 30 mL of toluene and was sonicated for 30 min. DA (0.1 g) was added to the above solution and the mixture was stirred. Then, the mixture was refluxed for 10 hours under a static dry nitrogen atmosphere. After the silylation reaction, the product (APTMS-DA) was collected by centrifugation, washed thoroughly with toluene and ethanol and then dried at 60^°^C.

### 2.4. Synthesis of hybrid organic-inorganic luminescent materials

First, 10 mg of APTMS-DA sample was added to ethanol and stirred. Then, the same volume of TTA/ethanol and Phen/ethanol solution with the concentration of 0.03 mol/L and 0.01 mol/L, respectively was dropped into the above solution and stirred for 10 min. Finally, the EuCl_3_ solution was added into the above mixture. After the pH was adjusted to 6 ~ 7 with ammonium hydroxide, the solution was stirred for 5 h at room temperature. The APTMS-DA hybrid luminescent material was collected and washed several times with ethanol to remove the dye until no luminescence was observed in the washing solution. The final deposit was dried at 60^°^C. The preparation of ETP-DA was similar to the method described above.

### 2.5. Preparation of composite film

The products obtained in the previous step and PET were vacuum dried at 80^°^C for 24 h to remove moisture. Then, the blending of ETP-APTMS-DA and PET were melt using a mini-twin screw extruder (HAAKE Rheomex PTW16). The content of ETP-APTMS-DA was 0.1%, 0.2% and 0.3% by weight. PET/ETP-APTMS-DA composite films were prepared using a hot press.

### 2.6 Characterization of materials

**Scheme 1:**
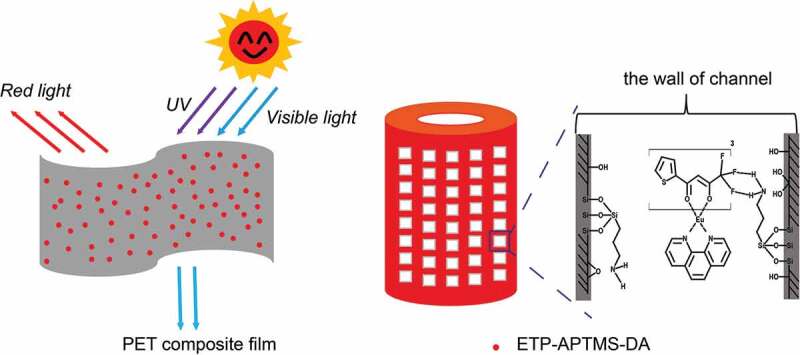
Possible UV-protection mechanism for the PET composite film

The morphologies of the samples were observed via field emission scanning electron microscopy (SEM, JSM 7500 F). The composition and crystal structure of the samples were characterized by X-ray diffraction (XRD, D8 Advance). The functional groups of the samples were explored via Fourier transform infrared spectrometry (FTIR, Nicolet 6700). The structure and the attachment modes to the diatomite surface of the siloxane molecules were investigated by nuclear magnetic resonance (^29^Si NMR, Bruker Avance III HD 400 MHz). Optical absorption spectra were recorded with an ultraviolet and visible spectrophotometer (UV-vis, Lambda750). The luminescence properties of the samples were studied with a fluorescence spectrometer (FLS 980-STM). The information on chemical bonds of the samples were investigated by X-ray photoelectron spectroscopy (XPS, ESCALAB 250Xi). Elemental analysis was carried out via inductively coupled plasma emission mass spectrometry (ICPMS, Agilent 7800). The specific surface area and pore size distribution of the samples were measured with an automatic specific surface area and pore analyzer (Tristar3000). A possible UV-protection mechanism of PET composite film is shown in Scheme1.

## 3. Results and discussion

### 3.1. Structural and chemical characterization of diatomite

**Figure 1.**
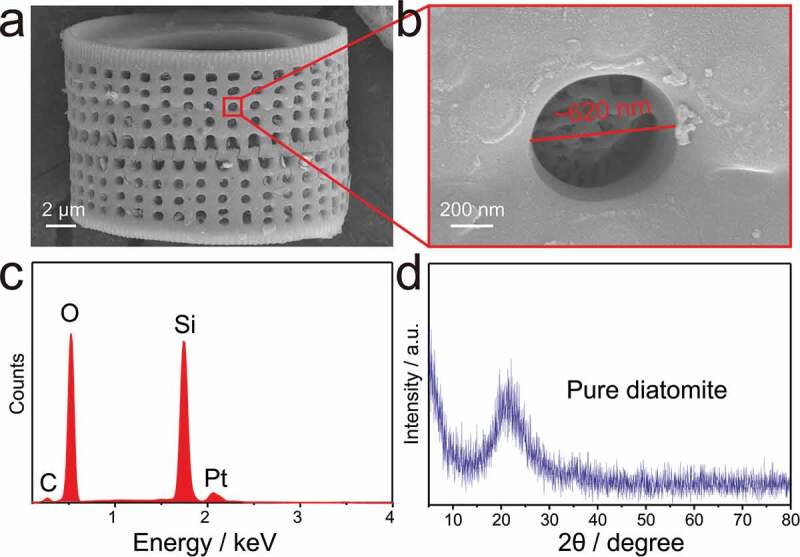
SEM images of (a) single frustule structure of purified diatomite and (b) close-up view of the porous structure. (c) EDS spectrum of chemical composition. (d) XRD pattern of diatomite silica

Figure 1(a) shows the characteristic structure of porous silica particles of diatomite. It can be clearly observed that the diatomite is composed of two halves interspersed and interlocking with each other. Meanwhile, there are regularly arranged rows of round holes with a diameter of 500–700 nm in the frustule. A detailed SEM view of the porous structure is shown in Figure 1(b). It is found that each hole has a self-growing entangled fibrous structure. Energy-dispersive X-ray spectroscopy (EDS) reveals that Si, O are the main elements of DA along with a small amount of C demonstrating that the DA mainly consists of silica with some organic impurities. Additionally, Pt is caused by Pt spray. The XRD pattern of pure DA shows a broad peak of amorphous SiO_2_ centred at 21°, which is consistent with that of diatomite in previous work [26,28]. Therefore, the diatomite material is primarily composed of the amorphous porous silica.

### 3.2. Organic Eu^3+^-complex anchored at porous diatomite channels through covalent bonding

**Figure 2.**
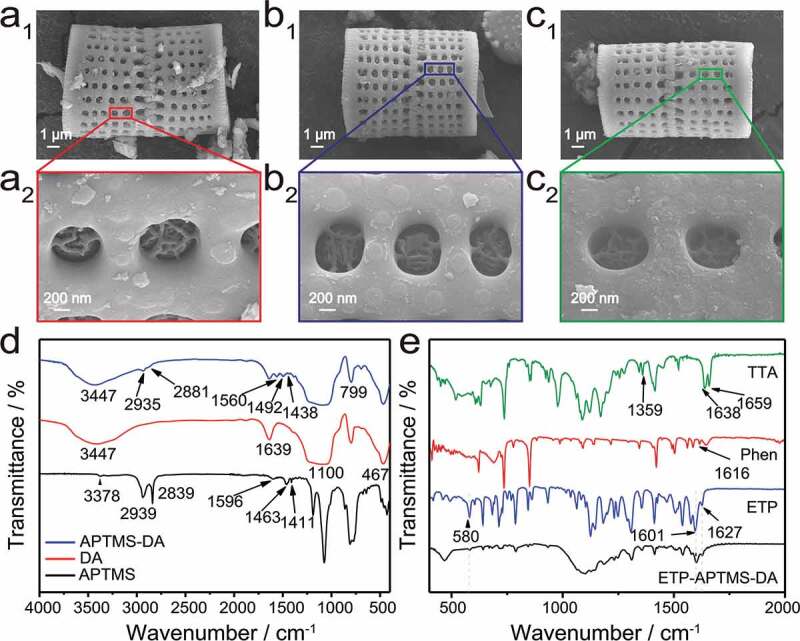
SEM images of (a_1_, a_2_) APTMS-DA, (b_1_, b_2_) ETP-DA and (c_1_, c_2_) ETP-APTMS-DA. FTIR of (d) DA, APTMS and APTMS-DA with (e) pure ETP and ETP-APTMS-DA

Figure 2(a–c) shows SEM images of DA, APTMS-modified DA, and ETP-APTMS-DA, respectively. By comparison with the original diatomite, it can be found that the morphology and size of the APTMS-modified sample have not changed obviously. Although the organosilane layer was not observed in the SEM image, FTIR and NMR data confirmed the presence of APTMS on the surface of DA. Meanwhile, the ETP-DA and ETP-APTMS-DA have no obvious changes also in morphology and pore structure compared to pure DA. However, FTIR and luminescence data also prove that the ETP complexes exist on the surface of DA and APTMS-DA samples. UV-vis and N_2_ adsorption-desorption results further demonstrate that the organic Eu^3+^-complex is encapsulated in the pores of the porous material.

As shown in Figure 2(d), FTIR results of the APTMS-DA provide the evidence for the existence of the organic moieties on the DA surface, compared to DA and APTMS used as the controls. The Si-O-Si framework of the diatomite silica presents the following absorbance bands: Si-O asymmetric stretching vibrations at 1100 cm^−1^, Si-O symmetric stretching vibrations at 799 cm^−1^, and Si-O-Si bending vibration at 467 cm^−1^. The hydroxyl (-OH) peaks at the range of 3100–3750 cm^−1^ and 1639 cm^−1^ present the stretching vibration and bending vibration, respectively, which derived from the physical adsorption water and the free hydroxyl groups on the surface area of the DA [27]. Actually, the existence of the free hydroxyl groups enables the functionalization of DA with APTMS. For APTMS, the peaks at wavenumber of 3378 and 1596 cm^−1^ can be attributed to the stretching and bending vibrations of primary amine. Moreover, the bands at 2939 (asymmetric stretching), 2839 (symmetric stretching), 1463 and 1411 cm^−1^ correspond to CH_2_ groups. The FTIR spectrum of APTMS-DA is similar to that of the DA. Whereas the appearance of C-H vibration at 2935, 2881, 1492 and 1438 cm^−1^and the N-H bending mode at 1560 cm^−1^ both prove the existence of organic moieties on the surface of the diatomite after modification. Therefore, it can be concluded that the surface of DA has been successfully modified by APTMS.

Through the FTIR analysis of the hybrid materials of ETP and ETP-APTMS-DA composites, the chemical bonds and functional groups were characterized in detail to prove the presence of ETP complexes in the APTMS-DA, which benefits to the FTIR results shown in Figure 2(e) that distinguishes the spectral changes of Phen and TTA in the range of 500–2000 cm^−1^ after the addition of Eu^3+^-complex to APTMS-DA. The absorption peaks of TTA at 1659 cm^−1^, 1638 cm^−1^ and 1359 cm^−1^ correspond to the vibration absorption peaks of C = O. The absorption peaks of Phen at 1616 cm^−1^ represents the vibration absorption peaks of C = N. Compared with TTA, the absorption intensity of C = O bond in ETP decreases and the absorption peak of C = O shifts to 1627 cm^−1^. Meanwhile, a new peak appears at 580 cm^−1^, which can be assigned to the stretching vibration of Eu-O [17]. These bands mentioned above indicate that a coordination bond is formed between C = O of TTA and Eu^3+^. Besides, the characteristic absorption peak corresponding to the C-N bond shifts from 1616 cm^−1^ to 1601 cm^−1^. Therefore, it can be speculated that a chemical bond is formed between Phen and Eu, and then the ETP is successfully synthesized. The spectrum of ETP-APTMS-DA shows both characteristic bands of the ETP complex and APTMS-DA. Based on the above characterization data, ETP complex is successfully attached on the surface of APTMS-DA.

**Figure 3.**
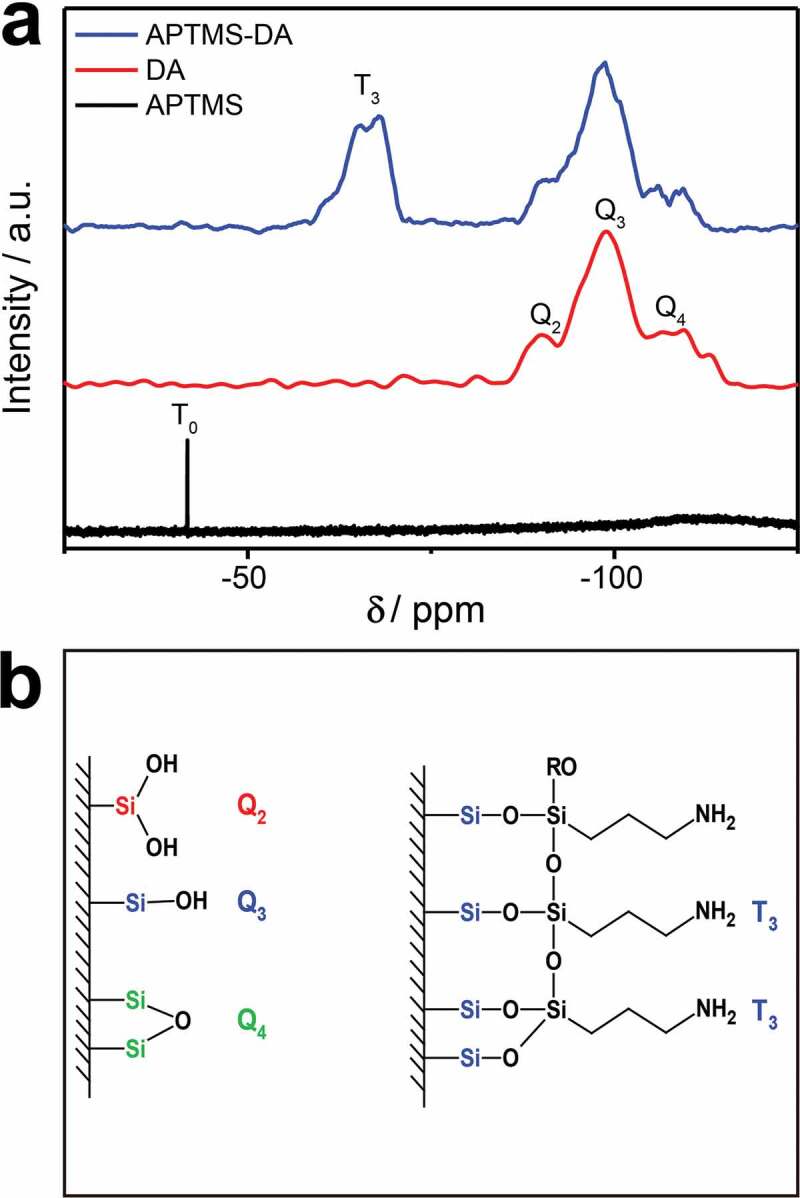
(a) ^29^Si NMR spectrum of APTMS and ^29^Si cross polarization magic-angle spinning NMR spectrum of DA and APTMS-DA. (b) The structural features of DA (left) and the proposed attachment mode of siloxane molecules on the DA surface (right)

Furthermore, ^29^Si NMR test was carried out and the results of the APTMS-modified DA (APTMS-DA) further confirm the successful reaction of APTMS with the surface of DA, compared to bare diatomite (DA) and APTMS used as the controls. The results are presented in Figure 3(a). The lower field resonance at −41.77 ppm in the APTMS can be attributed to a T_0_ structure in which no ethoxy group has been substituted. The three resonance signals at −90.14 ppm, −98.92 ppm and −109.53 ppm detected in the DA are corresponding to Q_2_, Q_3_ and Q_4_, respectively [36]. This is consistent with the DA structure with various types of bonds and the silanol groups, previously reported in the literature as shown on the left of Figure 3(b) [30]. For the APTMS-DA sample, a new separate peak (−67 ppm, corresponding to T_3_ structure) appears but the characteristic peak of APTMS disappears confirming that APTMS is not physically adsorbed on the DA surface and no excess siloxane molecules. The bonding modes of the APTMS to the DA surface are shown in Figure 3(b) (on the right). Consequently, APTMS is successfully bonded to the DA surface by chemical bonding.

Elemental analysis was used to determine the grafting ratio of APTMS grafted on the surface of DA and the related data are listed in Table 1. According to the changes of C, N and H content, elemental analysis also proves the successful modification of DA. The formula of the grafting ratio can be derived by the characteristics of the alkylation reaction and the calculation equation is as follows:

(1) $$M\;=\;1000\times W_{N}/M_{N}$$

**Table 1.** Elemental analysis of diatomite (DA) and APTMS modified DA (APTMS-DA)

Sample	Percent content (%)		
	N	C	H
DA	0.04	0.33	1.65
APTMS-DA	1.82	6.26	2.48

In the above formula, M corresponds to the effective molar density of APTMS grafted onto the DA surface. W_N_ is the percentage of N element of APTMS grafted on the surface of DA that can be obtained by subtracting the content of elemental N in DA from APTMS-DA. As can be seen from Table 1, the N content of APTMS-DA is 1.82%, while the N content of DA is 0.04% resulting in the W_N_ of 1.78%. The graft ratio of APTMS was calculated to be 1.27 mmol/g.

### 3.3. The anchored organic Eu^3+^-complex exhibits broadband UV absorption and internal energy conversion

**Figure 4.**
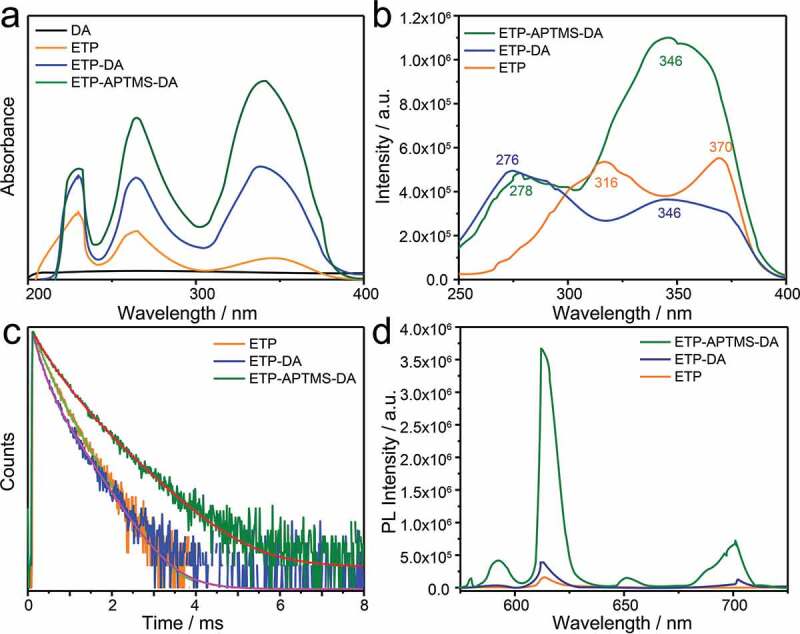
(a) UV-vis spectra of DA, ETP, ETP-DA and ETP-APTMS-DA. (b)Excitation, (d) emission spectra and (c) photoluminescence decay curves of pure ETP, ETP-DA and ETP-APTMS-DA

Figure 4(a) shows the UV-vis absorption spectra of DA, the pure ETP complex, ETP containing APTMS-modified DA and the original DA. The original DA material does not have a characteristic absorption band. The ETP itself has absorption bands at 230, 264, and 342 nm with the maximum centred at 230 nm corresponding to Phen ligand. Compared to the pure complex, both the ETP-APTMS-DA and ETP-DA all exhibit the absorption bands at 230, 264, and 339 nm with the maximum absorption at 339 nm from TTA ligand. The changes in peak intensity and position result from the Eu^3+^-complex anchoring into the channels of APTMS-modified as well as unmodified DA, so the polarity of the surrounding environment exhibits changes, which are similar to previous reports [19,20,23,24].

### 3.4. The anchored ETP in APTMS-DA can effectively increase the luminescence intensity and the lifetime of the organic Eu^3+^-complex

Luminescence properties of pure ETP, ETP-DA and ETP-APTMS-DA samples are shown in Figure 4(b). The excitation spectra were monitored with the maximum emission wavelength of 613 nm. It can be observed that in contrast to the pure ETP, the excitation bands of the organic Eu^3+^-complex in ETP-DA and ETP-APTMS-DA were broadened and shifted toward shorter wavelength, which can be attributed to the changes in the ligand environment. This is also proved by the UV-vis absorption spectra. To compare the performance of different luminescent samples, the emission spectra were obtained using their maximum excitation absorption band under the same condition. The result of PL spectra (Figure 4(d)) shows that all the three samples exhibit a maximum emission band around 613 nm corresponding to the ^5^D_0_-^7^F_2_ emission which results in ‘europium red’ luminescence. The transition of Eu(III) ion (^5^D_0_-^7^F_2_, at around 613 nm) is well known to be sensitive to the coordination environment of the Eu(III) ion. When the interaction of the ETP complex with its local chemical environment is stronger, the ETP complex becomes more asymmetric and the intensity of transition becomes higher. The ^5^D_0_-^7^F_2_/^5^D_0_-^7^F_1_ emission intensity ratios of ETP, ETP-DA and ETP-APTMS-DA are 8.2, 8.3 and 8.6, respectively, which reflects the increase in the intensity of the ^5^D_0_-^7^F_2_ emission band and the decrease in other emission bands due to the increase in electric dipole interaction [23]. Meanwhile, the FWHM of the ^5^D_0_-^7^F_2_ transition of the ETP and ETP-APTMS-DA samples is 8.30 and 9.48, respectively. Therefore, the bandwidth of the emission spectrum of the complex with the modified DA is significantly different with that of the pure complex. Besides, PL intensities of the three samples are increased with the order, i.e., pure ETP < ETP-DA < ETP-APTMS-DA. On the other hand, the emission quantum yields (Φ_tot_) are determined by the following procedure: when the emission wavelength is at 613 nm for all samples, the excitation wavelengths are verified at 370 nm, 346 nm and 346 nm for ETP, ETP-DA and ETP-APTMS-DA, respectively. The scanning wavelength range (200–800 nm) is used to get the absolute quantum yield. The emission quantum yields (Φ_tot_) are 5.19%, 6.41% and 17.52% corresponding to ETP, ETP-DA and ETP-APTMS-DA. With the similar sequence, the quantum yield of the ETP-APTMS-DA is higher than that of others. The changes can be rationalized on the basis of the interaction between NH_2_ groups and the ETP. Similar phenomenon and conclusion also are observed in other study [23].

The luminescence decay curves of Eu^3+^ ions related to ^5^D_0_-^7^F_2_ transitions in ETP, ETP-DA and ETP-APTMS-DA samples are shown in Figure 4(c). As seen in Figure 4(c), the decay traces were fitted using a polynomial Y(t) in equation (2):

(2) $$Y\left(t\right)\;=A+B_{1}exp\left(-t/\tau_{1}\right)+B_{2}exp\left(-t/\tau_{2}\right)$$

Luminescence decay components and their lifetime values are listed in Table 2. Although the fitting function, as shown in equation (2), is a polynomial, the curves in Figure 4(c) are completely smooth, which indicates that the essential structure to determine the photophysical properties in ETP, ETP-DA and ETP-APTMS-DA are unique, i.e., the complex structure of Eu^3+^ ions with TTA and Phen (i.e. ETP). The different decay behaviours in different samples are due to the existing vicinity difference of Eu^3+^ complex in ETP, ETP-DA and ETP-APTMS-DA. It can be seen that the lifetime of ETP-APTMS-DA is longer than ETP and ETP-DA. Indeed, the interaction between the complex and the functional group grafted on the DA surface changes the intensity and relaxation time of the molecular vibration. The short lifetime of ETP-DA can be speculated to relate to the quenching influence of O-H groups on the surface of DA on the complex [19,20,22–24].

**Table 2.** The luminescence decay components and lifetime values of ETP, ETP-DA and ETP-APTMS-DA

Samples	ETP	ETP-DA	ETP-APTMS-DA
A	0.96	0.96	1.90
B_1_	1548.33	2112.52	1776.80
B_2_	1579.10	885.31	1083.34
τ_1_ (ms)	0.19 (29.90%)	0.16 (44.17%)	0.36 (42.04%)
τ_2_ (ms)	0.44 (70.10%)	0.49 (55.83%)	0.80 (57.96%)

**Figure 5.**
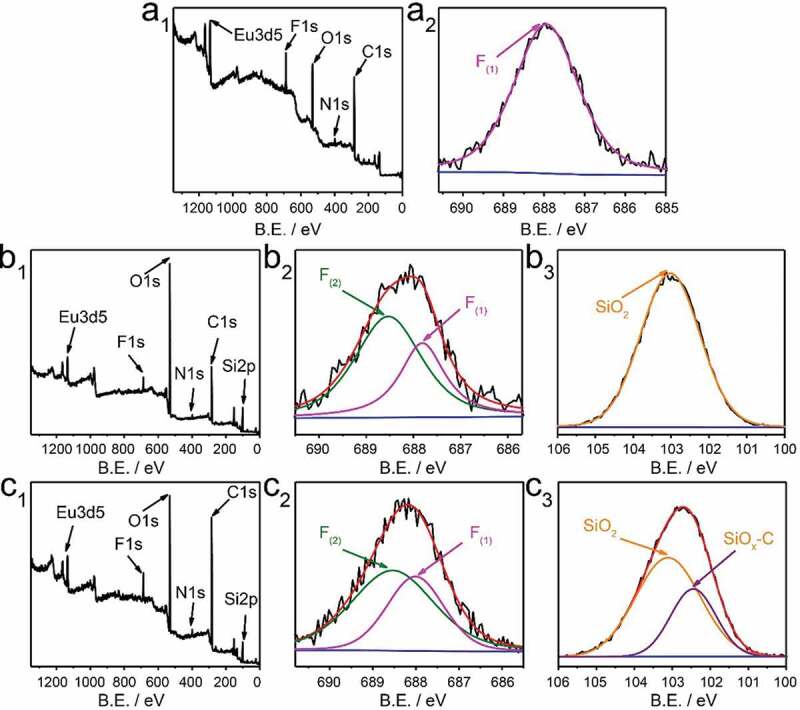
XPS survey spectra (a_1_, b_1_, c_1_), high-resolution XPS spectra of silicon F1s survey (a_2_, b_2_, c_2_), and Si2p (b_3_, c_3_). (a_1_, a_2_), (b_1_-b_3_) and (c_1_-c_3_) correspond to of ETP, ETP-DA and ETP-APTMS-DA, respectively. B.E. stands for binding energy

As shown in Figure 5, in order to explain the mechanism of enhanced luminescence performance of ETP-APTMS-DA, X-ray photoelectron spectroscopy (XPS) was measured to explore the chemical bonding of ETP in diatomite channels. The survey spectrum (Figure 5a_1_-c_1_) displays the signal of the Eu3d and the peaks of F1s, O1s, N1s, and C1s, which completely demonstrates the presence of TTA and Phen ligands. High-resolution XPS F1s spectrum of pure ETP is single-peak fitting, while the data of ETP-DA and ETP-APTMS-DA are well fitted by a combination of two peaks. The electron binding energy of the pure ETP complex is 687.97 eV corresponding to C-F. The F1s of the ETP-DA has two different binding energies, 687.82 and 688.52 eV, respectively. Similar to the ETP-DA, the F atom in ETP-APTMS-DA has two forms and the binding energy of the two decomposed peaks are 688.02 and 688.52 eV, respectively. The new peaks can be attributed to the charging effect of fluorine atoms [37]. Based on these differences of binding energy, the ETP and the surroundings are interacted with each other through the F atom of the complex, the hydrogen of -OH group and -NH_2_ group on the DA surface [23], which explains the blue shift of the UV-vis and excitation spectra. For the Si2p spectrum, the ETP-DA has the single peak with the energy of 103.12 eV related to SiO_2_ network structure), while the ETP-APTMS-DA has another lower energy of 102.47 eV corresponding to SiOx-C. This result also proves that APTMS successfully modified DA.

The contents of the Eu^3+^ ions in different samples were obtained by ICPMS measurement, to be 0.58% for ETP-DA and 1.48% for ETP-APTMS-DA, which reflects the strength of the interaction between ETP complex with APTMS-DA is stronger than that between ETP complex with DA.

**Figure 6.**
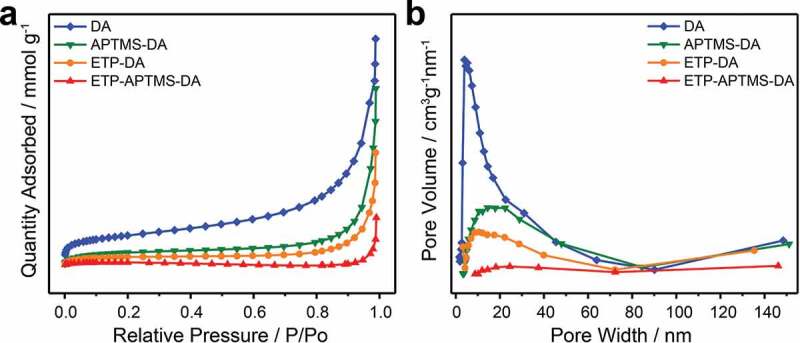
(a) N_2_ adsorption–desorption isotherms and (b) the pore size distribution curves of DA, APTMS-DA, ETP-DA, and ETP-APTMS-DA

The evaluations of channel size were measured to confirm that the anchored organic Eu^3+^-complex has been incorporated to porous channels of DA. The N_2_ adsorption-desorption isotherm and its BJH pore size distribution for DA, APTMS-DA, ETP-DA, and ETP-APTMS-DA are shown in Figure 6. The values of estimated BET specific surface area (SA) and BJH pore volume (PV) of DA, functionalized DA and their composites with ETP complexes are also summarized in Table 3. As expected, compared with pure diatomite materials, APTMS-functionalized DA materials have a smaller specific surface area and a smaller pore volume, which also confirms the successful functionalization of diatomite. Besides, the pore diameter decreases after surface modification due to the functional group covering the surface of the pore. When the ETP complex was incorporated to the APTMS-DA, smaller specific surface area and pore volume have been observed compared to APTMS-functionalized DA and pure diatomite. This is probably because of the presence of the organic complex in the channels of diatomite, similar as that Malba et al. observed [22]. Therefore, it can be concluded that the ETP complexes are encapsulated to APTMS-modified mesoporous diatomite material.

**Table 3.** Pore structure parameters of DA, APTMS-DA, ETP-DA, and ETP- APTMS-DA, derived from N_2_ adsorption–desorption isotherms

Samples	DA	APTMS-DA	ETP-DA	ETP-APTMS-DA
SA (m^2^g^−1^)	21.29	9.26	6.06	2.38
PV (cm^2^g^−1^)	0.06	0.04	0.03	0.01

### 3.5. The UV protection of PET composite films was studied

**Figure 7.**
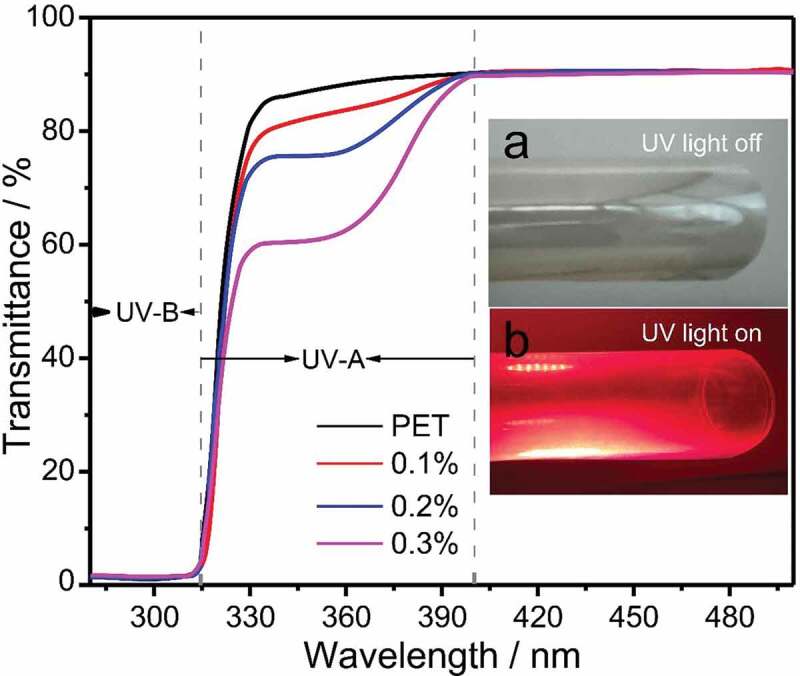
UV transmission curves of PET/ETP-APTMS-DA composite films. Inset shows 0.3% ETP-APTMS-DA composite film (a) under visible and (b) under UV illumination

PET composite film can not only absorb ultraviolet rays for protection but also utilize the UV light by converting UV light to emission energy (see Figure 7 inset). Therefore, the film may be used to protection of photovoltaic devices. Figure 7 shows the UV transmission spectra of pure PET film and different ETP-APTMS-DA doped PET composite films. It can be observed that the absorption edge of the pure PET film is only about 334 nm, while the absorption edge around 334–400 nm for PET/ETP-APTMS-DA composite film is due to the characteristic absorption peak of ETP complex. Additionally, the absorption intensity increases after the addition of the ETP-APTMS-DA material to the PET and furthermore, the absorption intensity enhances with the amount of ETP-APTMS-DA increasing. Pure PET films show a high transparency of about 90% while the films prepared by mixing ETP-APTMS-DA with PET possess the similar transmission. The film has a UV absorption of about 40% with the addition of 0.3% ETP-APTMS-DA.

## 4. Conclusions

In this work, we put effort to explore the new usage of porous natural material, diatomite, for high efficient luminescent materials and further for UV protection in soft host. The most important innovation is the successful synthesis of organic Eu^3+^-complex-anchored porous diatomite channels that enable UV protection and downconversion in hybrid PET-matrix film. We found that ETP anchored at APTMS-modified DA surface, and exhibited the long lifetime and the high luminescence intensity. The XPS and UV-vis absorption spectra demonstrated that the emission properties of the organic Eu^3+^-complex were greatly influenced by its vicinity interactions in ETP complex and APTMS-DA. Generally, the photoluminescence properties of ETP complexes anchored in APTMS-DA were improved considerably in comparison to other two samples. On the other hand, the transparency of pure PET film is approximately 90% at 500 nm without significant UV absorption for UV-A, whereas the UV-A absorption of ETP-APTMS-DA doped PET film is ~40%. Therefore, ETP-APTMS-DA can be used as an excellent UV blocker in PET transparent films. As expected, this UV-protection flexible film will potentially apply for the large area flexible polymer solar cell to enhance efficiency and to extend service life.
